# A MZB Cell Activation Profile Present in the Lacrimal Glands of Sjögren’s Syndrome-Susceptible C57BL/6.NOD-*Aec1Aec2* Mice Defined by Global RNA Transcriptomic Analyses

**DOI:** 10.3390/ijms23116106

**Published:** 2022-05-29

**Authors:** Ammon B. Peck, Cuong Q. Nguyen, Julian L. Ambrus

**Affiliations:** 1 Department of Infectious Diseases and Immunology, College of Veterinary Medicine, University of Florida, P.O. Box 100125, Gainesville, FL 32610, USA; nguyenc@ufl.edu (C.Q.N.); jambrus@buffalo.edu (J.L.A.J.); 2 Division of Allergy, Immunology and Rheumatology, SUNY Buffalo School of Medicine, 875 Ellicott Street, Buffalo, NY 14203, USA

**Keywords:** Sjögren’s syndrome, C57BL/6.NOD-*Aec1Aec2* mouse, marginal zone B (MZB) cells, RNA transcriptome microarray, Notch2 signal transduction pathway, Rho-GTPases, Dock proteins, B cell receptor (BCR)

## Abstract

The C57BL/6.NOD-Aec1Aec2 mouse has been extensively studied to define the underlying cellular and molecular basis for the onset and development of Sjögren’s syndrome (SS), a human systemic autoimmune disease characterized clinically as the loss of normal lacrimal and salivary gland functions leading respectively to dry eye and dry mouth pathologies. While an overwhelming majority of SS studies in both humans and rodent models have long focused primarily on pathophysiological events and the potential role of T lymphocytes in these events, recent studies in our murine models have indicated that marginal zone B (MZB) lymphocytes are critical for both development and onset of SS disease. Although migration and function of MZB cells are difficult to study in vivo and in vitro, we have carried out ex vivo investigations that use temporal global RNA transcriptomic analyses to track early cellular and molecular events in these exocrine glands of C57BL/6.NOD-Aec1Aec2 mice. In the present report, genome-wide transcriptome analyses of lacrimal glands indicate that genes and gene-sets temporally upregulated during early onset of disease define the Notch2/NF-kβ14 and Type1 interferon signal transduction pathways, as well as identify chemokines, especially Cxcl13, and Rho-GTPases, including DOCK molecules, in the cellular migration of immune cells to the lacrimal glands. We discuss how the current results compare with our recently published salivary gland data obtained from similar studies carried out in our C57BL/6.NOD-Aec1Aec2 mice, pointing out both similarities and differences in the etiopathogeneses underlying the autoimmune response within the two glands. Overall, this study uses the power of transcriptomic analyses to identify temporal molecular bioprocesses activated during the preclinical covert pathogenic stage(s) of SS disease and how these findings may impact future intervention therapies as the disease within the two exocrine glands may not be identical.

## 1. Introduction

Autoimmunity is generally recognized as a multi-step process initiated by environmental triggers that activate an innate inflammatory reaction which progresses to an adaptive immune response targeting a critical biological process in genetically or physiologically predisposed hosts whose dysfunction eventually results in an overt clinical pathology [1,2,3,4,5,6]. Autoimmune diseases, therefore, are considered to have both an early covert disease phase and a late overt disease phase. Unfortunately, patients most often present in clinic only after the adaptive immune phase is active and irreversible pathology has occurred. This scenario is especially true for Sjögren’s syndrome (SS), a highly debilitating, yet fascinating systemic autoimmune disease marked primarily by leukocytic infiltrations of the salivary and lacrimal glands with the concomitant loss of exocrine secretion leading to clinical symptoms of severe dry mouth and dry eye problems (reviewed in [7]). Furthermore, SS is a systemic autoimmune disease having a skewed prevalence towards women, potentially capable of involving multiple organ systems, exhibiting a wide variety of symptoms, and associated with lymphomagenesis [8]. An additional complication is the time between disease onset and a correct diagnosis which ranges from 4 to 10 or more years [7,9,10].

B cells and their cellular products, together with their ability to act as antigen-presenting cells and secrete auto-antibodies, are important factors in rheumatoid autoimmune diseases in general (reviewed in [11,12,13]), and Sjögren’s syndrome (SS) specifically [7,14,15,16]. Over the past couple decades, we have studied a number of rodent models, including NOD/ShiLtJ [17] C57BL/6.NOD-*Aec1Aec2* [18], (C57BL/6.NOD-*Aec1Aec2 X* C57BL/6J)F1 recombinant inbred (RI) lines [19], and C57BL/6.*Il14a* transgenic (TG) [16,20] NOD-^−/−^ [21], NOD-*scid* [22], NOD-*Ifng*^−/−^ [23], NOD-*IL4*^−/−^ [24], NOD-*Stat6*^−/−^ [25], NOD-*C3*^−/−^ [20,26], and NOD-*Il17a*^−/−^ [27]. In those strains that develop a SS-like pathology, the disease appears spontaneously, develops temporally, and is characterized by: (a) aberrant proteolytic enzyme activity, (b) progressive loss of saliva and tear flow rates accompanied by increased protein content, (c) decline in amylase and carbonic anhydrase activities, and (d) appearance of autoantibodies, all manifestations dependent on the presence of B cells and occurring concomitantly with increasing glandular leukocyte infiltrates. Recent studies have also shown a direct correlation between an upregulated expression of IL14α and late-stage B cell lymphomagenesis [28,29] as well as the fact that full development of disease is a multi-phase process involving an innate inflammatory response, an adaptive response, and in some individuals, a late lymphomagenesis phase and/or organ involvement of kidneys and lungs. This progression of autoimmune pathologies in these models is significant when compared against sex- and aged-matched mice that do not develop SS-like disease despite developing occasional glandular infiltrations [30,31].

Studies in both BAFF [32,33,34] and B6.*Il14α* transgenic (TG) mice [28,29] have provided strong evidence that MZB cells are a critical cell population for onset and early disease phase development. Elimination of the MZB cell population or blocking the lymphotoxin activity required for MZB cell ontogeny [35] were shown to prevent development of SS-like disease, including lymphomagenesis. In support of these findings, our most recent genome-wide transcriptome studies of ex vivo salivary glands from the C57BL/6.NOD-*Aec1Aec2* mice [36,37] have provided further evidence that MZB cells are recruited to the salivary glands during early-stage disease, thus apparently establishing an environment conducive for a subsequent destructive T-cell-mediated cytotoxic autoimmune attack. To follow up on these results, we have now carried out a similar transcriptome analysis using ex vivo lacrimal glands from our C57BL/6.NOD-*Aec1Aec2* mice. The study has permitted a direct data comparison between the two glandular tissues as the tissues were derived from the same mice and the gene sets compared data from identical aptamers/probes. Results reveal overall similar, but not necessarily identical, profiles.

## 2. Results

### 2.1. Early and Temporal Transcriptome Expressions in Lacrimal Glands of C57BL/6.NOD-Aec1Aec2 Mice Identify MZB Cell Involvement in SS Pathology but with Potentially Unique BCRs

**Figure 1:**
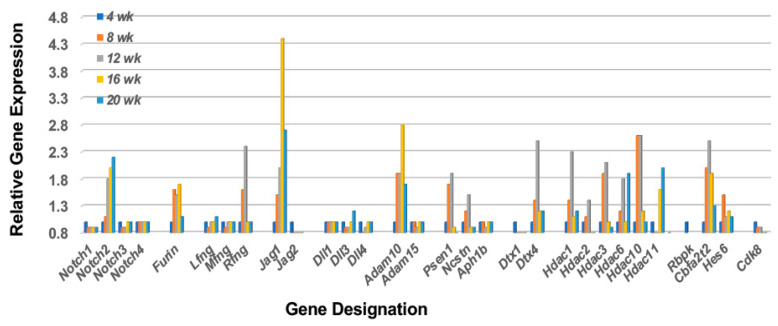
Prolonged upregulated activation of the Notch2 signal transduction pathway in lacrimal glands prior to and during onset of the adaptive response. Notch2 is a transmembrane protein receptor whose extracellular portion is post-translationally modified in the Golgi bodies by furin and fringe molecules (i.e., Lfng, Mfng, or Rfng) prior to its insertion into the cellular membrane, thus enhancing subsequent MZB cell functions. On activation by a Notch2 ligand (i.e., Jagged and/or Delta molecules), the Notch2 molecule undergoes sequential cleavage first by Adam10 and then by γ-secretase, thereby releasing the cytoplasmic protein Dtx (an E3 ubiquitin ligase), as well as the Notch intracellular domain (NICD) that gets transported into the nucleus. In the nucleus, NICD displaces corepressors, including HDACs (histone deacetylase complex), allowing direct interaction with Rbpk to activate Hes, thus driving Notch2-regulated gene transcription. Downregulation of the Notch2 pathway occurs through phosphorylation of NCID by Cdk8 that initiates polyubiquitination and proteasome degradation. In this model, however, while the Notch2 pathway remains activated, Cdk8 exhibits, at best, minimal upregulated expression, suggesting the MZB cells may persist and over time differentiate from their effector function to their APC function for invoking the adaptive response.

In support of the hypothesis that MZB cells are strongly involved in the early immune attack on the lacrimal glands of C57BL/6.NOD-*Aec1Aec2* SS-susceptible (SS^S^) mice, our temporal global transcriptome data reveal a unique upregulated Notch2 signal transduction profile, as presented in Figure 1. Based on the specific set of upregulated genes concomitantly expressed, the cellular profile and signal transduction pathway defined is: *Notch2* >*Furin* > *Rfng* > *Jag1* > *Adam10* > *γSecretase* (*Psen1* and *Ncstn*) > *Dtx4* > *Hdac* > *Rbpk* > *Cbfat2* > *Hes6*, a pathway leading to Notch-specific gene transcription capable of activating multiple cell functions, including that of Nfkβ1 (nuclear factor kappa b subunit 1), Mapk14 (mitogen-activated protein kinase 14), and Infa1 (interferon type1). This early appearance also corresponds with the first observation of migration and infiltration of lymphocytes into the lacrimal glands noted histologically [38]. In addition, the transcriptome data reveal that MZB cells entering the lacrimal glands between 8 and 12 weeks of age are exhibiting both ontogenesis (increase receptor synthesis) and cell activation (increased Notch2 signal transduction). Furthermore, by 16 weeks of age, the transcriptome profile reveals a strong continued Notch2 profile, thereby suggesting a relatively prolonged activated state. While this overall profile corresponds with the onset of an inflammatory/innate response and initiation of early pathology within the lacrimal gland, respectively, it does not specify if this represents a single continuous cellular event or two time-dependent activities of multiple independent events. Interestingly, this lacrimal gland *Notch2* profile differs slightly from the *Notch2* gene profile seen in our recently published profile of salivary glands [36], specifically, (a) the lacrimal gland profile shows an upregulation of the *Furin*, *Rfng*, and *Jag1* gene set in the Notch2 pathway, while the salivary gland profile shows an upregulated *Furin*, *Mfng*, *Rfng*, and *Jag2* set of genes, and (b) the lacrimal gland profile indicates a strong upregulation of Deltex family gene *Dtx4*, while the salivary gland shows a strong upregulated *Dtx1* gene instead. Nevertheless, since these two gene sets encode for MZB cell receptors and proteins that regulate Type 1 interferon (IFN1) production, these data raise the possibility that different MZB cell subpopulations are emigrating to the two different exocrine glands and recognizing different autoantigens.

**Figure 2:**
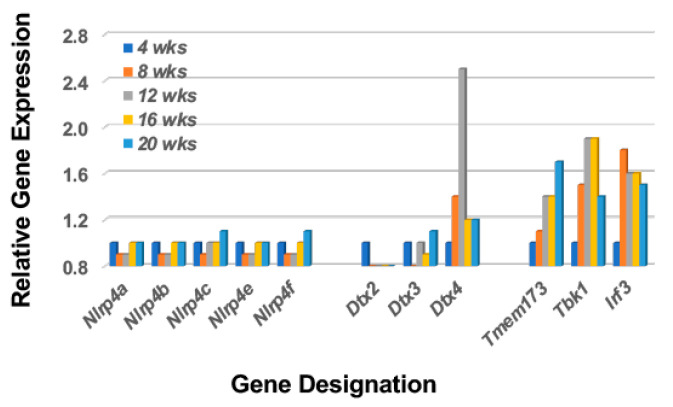
A transcriptome profile that predicts activation of the type 1 IFN response despite upregulation of *Dtx* expression. The transcriptome profile of the lacrimal glands of C57BL/6.NOD-*Aec1Aec2* mice indicate a lack of an upregulated *Nlrp4* gene expression and thereby a concomitant targeting of Tbk1 by Dtx4. This profile would explain the strong upregulation of the signal transduction pathway for Type 1 interferon and activation of the interferon-signature characteristic of SS.

A major function of the Notch2 pathway in stimulated MZB cells is to activate an IFN1 response, a critical activity for stimulating the interferon signature and subsequent SS disease. Regulation of Ifn1 synthesis is, in part, dependent on activation of Dtx4 (an E3 ubiquitin ligase) by Nlrp4. The Nlrp4 regulates Ifn1 synthesis by targeting Tbk1 (Tank-binding kinase 1) for degradation by Dtx4. Tbk1 degradation, therefore, is a critical component in down-regulation of Ifn1 in the activation pathway involving *Tmem173* (Sting) > *Tbk1* > *Irf3* (Interferon regulatory factor *3*). As shown in Figure 2, despite an upregulated *Dtx4* expression, none of the possible *Nlrp4* genes expressed in mice (i.e., *Nlrp4a*, *b*, *c*, *e* and *f*) exhibit temporal upregulated expressions, thereby permitting Tbk1 function to persist long-term. This profile, which is similarly seen in the salivary glands of C57BL/6.NOD-*Aec1Aec2* mice [37], predicts a strong and prolonged Ifn1 response in the lacrimal glands of these SS^S^ mice during the early disease state as well.

### 2.2. The Notch2 Signaling Pathway Predicts Activation of Mitogen-Activated Protein Kinase 14 (p38Mapk14α)

**Figure 3:**
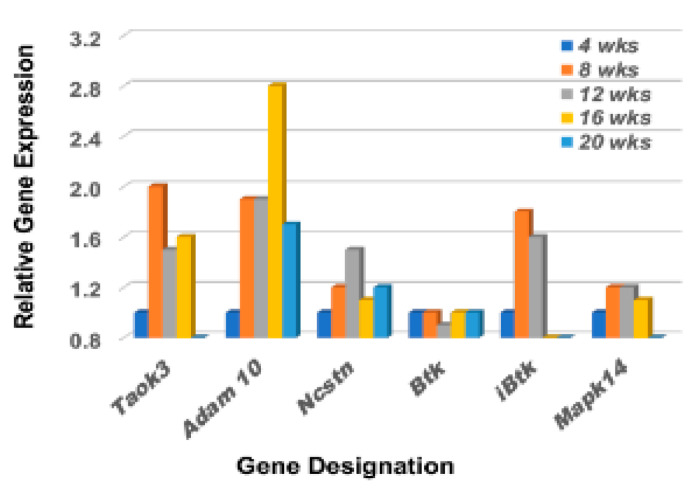
Differential expression profiles for genes encoding various factors involved in the upregulation of MZB cell signal transductions. Comparative transcriptome data showing the temporal expression of genes encoding factors critical in regulating the Notch2 signal transduction pathway of MZB cells, i.e., *Taok3* (TAO kinase 3), *Adam10*, *Ncstn* (Nicastrin), *Btk* (Bruton’s tyrosine kinase), *iBtk* (Inhibitor of Bruton’s tyrosine kinase), *Prp38* (Proline rich protein 38), and *Mapk14a* (Mitogen-activated protein kinase 14a) active in lacrimal glands of C57BL/6.NOD-*Aec1Aec2* mice starting at 8 wks of age.

Although each Notch2 pathway component is essential in dictating specific MZB cell characteristics and functions that form the final immune profile, there are five interesting critical factors of regulation that help determine MZB cell differentiation and activation. These are Taok3 (Tao kinase 3), Adam10 (A disintegrin and metallopeptidase domain), Ncstn (Nicastrin), Btk (Bruton’s tyrosine kinase), and Ibtk (Inhibitor of Btk) [39,40,41,42]. While Taok3 is a factor expressed in virtually all tissues, one function is to commit T1 B cells to a MZB cell fate by mediating surface expression and activation of Adam10 via the BCR and Notch2 ligand pathways. Adam10 is expressed on mature MZB cells, but not on follicular B cells, and activates Notch2 downstream signal transductions. Nicastrin, a member of the γ-secretase complex, also plays an important role in the transitioning of T2 B cells towards MZB cells by glycosylating γ-secretase. In contrast, Btk blocks T2 B cell differentiation into MZB cells, instead promoting the differentiation of T2 B cells into follicular B cells. In contrast, Ibtk suppresses Btk function. As presented in Figure 3, the genes encoding Taok3, Adam10, and Nicastrin are each upregulated in the lacrimal glands of C57BL/6.NOD-*Aec1Aec2* mice starting as early as 8 weeks of age. At the same time, the gene encoding Btk remains quiescent, apparently suppressed by an upregulated Ibtk, thus further supporting the promotion of T2B cell differentiation into MZB cells. Interestingly, *Taok3* gene expression reveals a different profile from *Adam10* and *Ncstn*, but this overall profile is strongly reflective of MZB cell activation of p38Mapk14 (a dimeric protein composed of p38β p38α which, when coupled, can activate multiple systems) in the lacrimal glands even though demonstrating a relatively weak temporal upregulation.

### 2.3. Limited Receptor Gene Expressions That Can Activate Early-Phase Immune Functions of MZB

**Figure 4:**
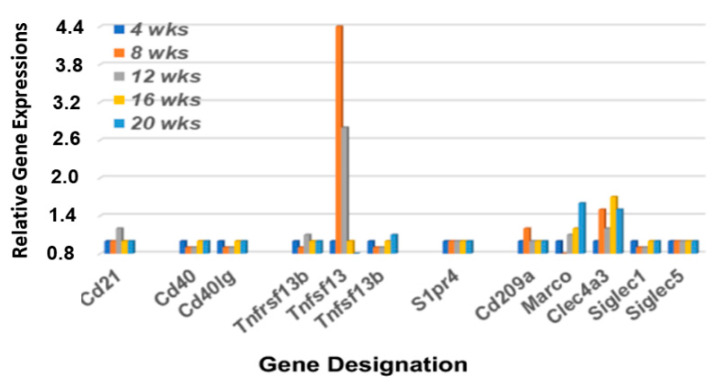
Differential expression profiles for genes encoding various receptors associated with MZB cell functions. Transcriptome data showing the temporal expression of MZB cell-associated receptor genes (*Cd21*, *Cd40*, *S1pr4*, and *Tnfrsf13b*), receptor ligands (*Cd40lg*, *Tnfsf13*, and *Tnfsf13b*) and macrophage receptors (*Cd209b*, *Marco*, *Clec4a3* and *Siglec1*) in lacrimal glands of C57BL/6.NOD-*Aec1Aec2* mice.

In addition to B cell receptors (BCRs) and Toll-like receptors (TLRs), MZB cells also express Cr2 (the complement receptor encoded by *Cd21*), CD40 (the receptor for Cd40-ligand encoded by *Cd40*), IL22r (the interleukin 22 receptor encoded by *Il22ra*), S1pr (the sphingosine-1-phosphate receptor encoded by *S1pr4*), and TACI (encoded by *Tnfrsf13b*) [43,44,45,46,47]. Furthermore, MZB cells can be activated by either MZ macrophages expressing SIGN-R1 (encoded by *Cd209a*), Marco (macrophage receptor with collagenous structure encoded by *Marco*), and DCIR (encoded by *Clec4a*), or by metallophilic macrophages expressing Cd169 (encoded by *Siglec1*). As shown in Figure 4, of these genes, only *Tnfsf13*, *Sign-R1* and *Clec4a3* exhibit upregulated expressions in the lacrimal glands of C57BL/6.NOD-*Aec1Aec2* mice that start around 8 wks of age, with *Marco* exhibiting an upregulated expression at a later age. These data also suggest an absence of metallophilic macrophages, since there is no upregulated *Cd209a* expression. Interestingly, this lacrimal gland profile contrasts with the profile reported for this gene set in salivary glands [36] where genes encoding for Cd21, S1pr4, Tnfrsf13b (Baff), Tnfsf13 (April), Marco, Cd209b, and Clec4a4 are each upregulated simultaneously at 16 weeks of age. Unexpectedly, neither *Cd40* nor *Cd40lg* are upregulated in the lacrimal gland, thus mimicking what we reported in the salivary glands of C57BL/6.NOD-*Aec1Aec2* mice. These data further support the concept that the B cell population in the exocrine glands are MZB cells and not B2 cells.

### 2.4. Receptor-Ligand Dependent Emigration of Immune Cells to the Lacrimal Glands

**Figure 5:**
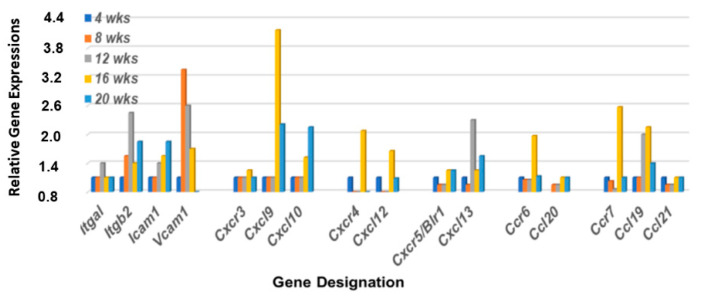
Differential expression profiles for genes encoding integrin, chemokine, and chemokine receptors critical for MZB cell emigration from the MZ regions. Transcriptomic data reveal that genes encoding LFA-1 subunits (*Itga1* and *Itgβ2*), together with *Cxcl13*, show a coordinated upregulated expression at 12 wks of age in the lacrimal glands of C57BL/6.NOD-*Aec1Aec2* mice. Chemokine receptors *Cxcr4* and *Ccr7*, together with their ligands *Cxcl12* and *Ccl19*, show strong upregulations at 16 wks of age, while chemokine receptor *Cxcr3* and chemokine ligands *Ccl20* and *Ccl21* exhibit no upregulated expressions.

An interesting phenomenon that distinguishes MZB cells from follicular B cells, particularly in mice, is the relatively non-circulatory state of MZB cells compared to the more robust migratory activity of follicular B cells in the normal state. As proposed by Lu and Cyster [48], this difference occurs because of the upregulated surface levels of integrin expressions on MZB cells, especially LFA-1. LFA-1 is a heterodimeric integrin, comprised of ItgαL and Itgβ2 subunits that, in a normal state, binds to ICAM-1 expressed in marginal zones (MZs). Development of splenic MZ areas during ontogeny requires lymphotoxin-dependent integrin-mediated induction of ICAM-1 and VCAM-1, with ICAM-1 being the primary receptor for LFA-1. High levels of integrin expression on MZB cells raises the threshold levels of chemokines required to dissociate MZB cells from splenic MZs for emigration to other tissue sites. As presented in Figure 5, our transcriptomic data reveal that genes encoding LFA-1 integrin subunit *Itgal* is minimally upregulated while integrin subunit *Itgb2* is highly upregulated, starting around 12 weeks of age. At the same time, expression of *Icam1* and *Vcam1* show opposing upregulated profiles from 8 to 20 wks of age, indicating development of an environment in the lacrimal glands suitable for capturing MZB cells during this time frame.

MZB cells also express multiple chemokine receptors, especially Cxcr3, Cxcr4, Ccr5, Ccr6, and Ccr7. Our previously published data [36,37] have shown that genes encoding these five chemokine receptors are highly upregulated in the salivary glands of C57BL/6.NOD-*Aec1Aec2* mice at 4 months of age, along with the genes encoding chemokine ligands *Cxcl9*, *Cxcl10*, *Cxcl12*, *Cxcl13*, *Ccl19*, and *Ccl21*. In contrast, chemokine receptor genes *Ccr1*, *Ccr2*, *Ccr3*, *Ccr8*, *Ccr9*, and *Ccr10* showed no upregulation in the salivary glands, suggesting limited or no involvement of neutrophils up to 20 wks of age. As presented in Figure 5, the gene expression profile for these chemokines in the lacrimal glands of C57BL/6.NOD-*Aec1Aec2* mice exhibit a different transcriptome profile with chemokine receptor gene *Cxcr4*, *Ccr6*, and Ccr7 showing a strong upregulation, *Cxcr3* and *Ccr5* a weak upregulation, and the chemokine ligands *Cxcl9*, *Cxcl10*, *Cxcl12*, and *Ccl19* a substantial upregulation.

### 2.5. Identification of Cell Emigration to the Lacrimal Glands in C57BL/6.NOD-Aec1Aec2 Mice

The temporal gene expression profile presented above strongly suggests an emigration of MZB cells in the earliest stages of SS development in the lacrimal glands of C57BL/6.NOD-*Aec1Aec2* mice. In addition, the upregulation of the Notch2 signal transduction pathway suggests this specific B cell population is undergoing both ontogenesis and cell activation followed by transcription supporting a relatively prolonged functional state. This profile also corresponds temporally with the onset of the inflammatory/innate response and initiation of the adaptive response within the lacrimal glands observed histologically [38]. One of the main manifestations of SS disease is the progressive periductal leukocytic infiltrations of the lacrimal and salivary glands, but while this emigration and expanding leukocytic infiltration is considered an important pathological event, its molecular basis remains poorly defined.

**Figure 6:**
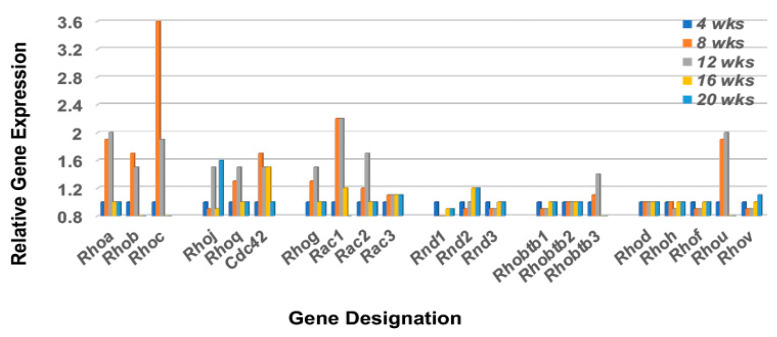
Transcriptomic profile of the Rho-GTP family of proteins in the lacrimal glands of C57BL/6.NOD-*Aec1Aec2* mice. Rho-GTPase family proteins are important in regulating the cellular homeostasis of the GTP<>GDP system. The Rho-GTP family consists of 21 members of which 16 are divided among 5 subfamilies (Rsf1, Rsf2, Rsf3, Rsf4 and Rsf5) with 5 unassigned (designated Rsfx). For cells in the non-activated state, Rho-GTP molecules are mostly associated with cellular membranes, but during cell activation the molecules dissociate to the cytoplasm by an as yet unknown mechanism.

Cell migration is highly dependent on at least three bio-entities: chemokine signaling, focal adhesion assembly, and Rho-GTP family protein activations. Although we have presented a chemokine > chemokine receptor profile above and have previously published the molecular composition of the focal adhesion(s) activated in the lacrimal glands of C57BL/6.NOD-*Aec1Aec2* mice [49], no profiling of Rho-GTP gene expressions in this SS^S^ model have yet been performed. In a quiescent state, Rho-GTP proteins mostly localize to cellular membranes but dissociate from these membranes during initiation of migration. In mice, the Rho-GTP family consists of 21 members subdivided into several subfamilies: 11 Rho proteins, 3 Rnd proteins, 3 Rac proteins, 3 Rhobtb proteins and Cdc42 [50]. Interestingly, the profile of these molecules in the lacrimal glands indicates that seven *Rho* genes (a, b, c, g, j, q, and u), two *Rac* genes (1 and 2), one Rhobtb gene (3), and the Cdc42 gene are upregulated, and in each case, upregulated at 8 to 12 weeks of age (Figure 6).

**Figure 7:**
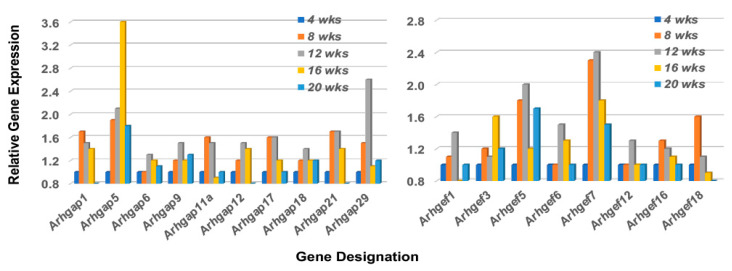
Temporal gene expressions for the Rho-GTP *Arhgap* and *Arhgef* gene family members upregulated in the lacrimal glands of C57BL/6.NOD-*Aec1Aec2* mice during onset of SS. Transcriptome data indicating the temporal expressions of the 8 of 23 *Arhgap* genes (left), and 8 of 17 *Arhgef* genes (right) found to be upregulated in the lacrimal glands.

**Figure 8:**
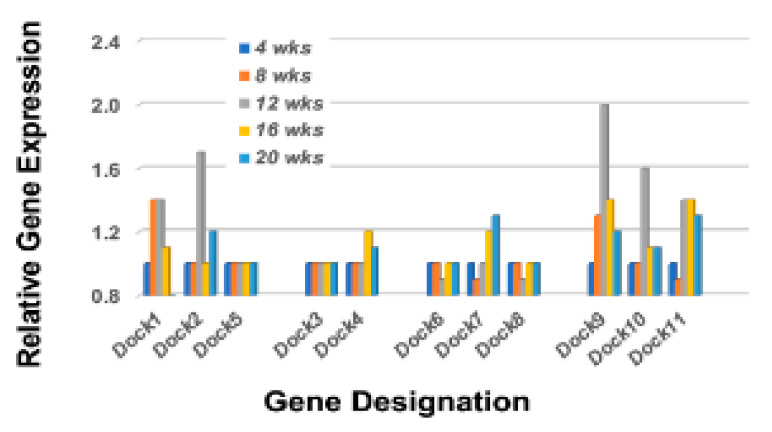
Temporal gene expressions for the Rho-GTP *Dock* gene family members. Transcriptome data showing the temporal expressions of the *Dock* genes upregulated in the lacrimal glands of C57BL/6.NOD-*Aec1Aec2* mice. Two genes, *Dock1* and *Dock9*, exhibit an upregulated expression starting at 8 wks of age, while *Dock2*, *Dock10*, and *Dock11* exhibit an upregulated expression starting at 12 wks of age.

The Rho-GTP proteins are further divided into two functional classes, those that are incapable of hydrolyzing GTP (i.e., Rhoh and Rnd subfamily members that constitutively bind GTP), and those that hydrolyze GTP through an opposing equilibrium of Rho-specific guanine nucleotide exchange factors (GEFs) and GTPase-activating proteins (GAPs) [50,51]. GEFs activate Rho-GTPases by exchanging a bound GDP with a GTP, while GAPs inactivate Rho GTPases vis-a-vis GTP by catalysis. Temporal gene expression profiles for GAP and GEF molecules in the lacrimal glands of C57BL/6.NOD-*Aec1Aec2* mice are summarized in Figure 7 left and right, respectively. These gene expressions again reveal a restricted activation with ten of the 23 *Arhgap* gene family members (1, 5, 6, 9, 11a, 12, 17, 18, 21, and 29) and eight of the 17 *Arhgef* gene family members (1, 3, 5, 6, 7, 12, 16, and 18) upregulated. In addition to the Arhgef protein family, a second subset of Rho-GEF proteins are the eleven DOCK (Dedicator of cytokinesis) family proteins [52,53,54]. DOCK molecules, also known as CZH proteins, possess functional CZH2 domains that promote the exchange of GDP to GTP in the formation of Rho-GTPases. Based on sequence homologies represented within the eleven identified DOCK molecules, this molecular family of proteins is currently divided into four major subgroups, (A) Dock1, 2 and 5, (B) Dock3 and 4, (C) Dock6, 7, and 8, and (D) Dock9, 10 and 11. Besides playing an important role in multiple cellular functions, an additional critical function is cell migration, especially Dock2, 9, 10, and 11. As presented in Figure 8, the temporal gene expression profiles of the Dock molecules in the lacrimal glands of C57BL/6.NOD-*Aec1Aec2* mice is consistent with this concept, as highest upregulated temporal expression activity is in *Dock 2*, *9*, *10*, and *11* occurring concomitantly with development and onset of SS in this mouse model. The upregulation of *Dock1* is also consistent with glandular structural changes taking place at 8–12 weeks of age [55,56]. Also of interest is the fact that development and accumulation of MZB cells in MZs, followed by their subsequent migrations, appear to require the *Dock2 > Rho-GEF > Pyk2* molecular pathway [50,53]. Furthermore, the recently published work from Gotoh et al. [54] has shown that Dock2 is indispensable for the co-migration of plasmacytoid dendritic cells (pDC), a highly relevant fact considering the extensive type 1 IFN-signature seen in exocrine glands of both human and mouse SS diseases.

**Figure 9:**
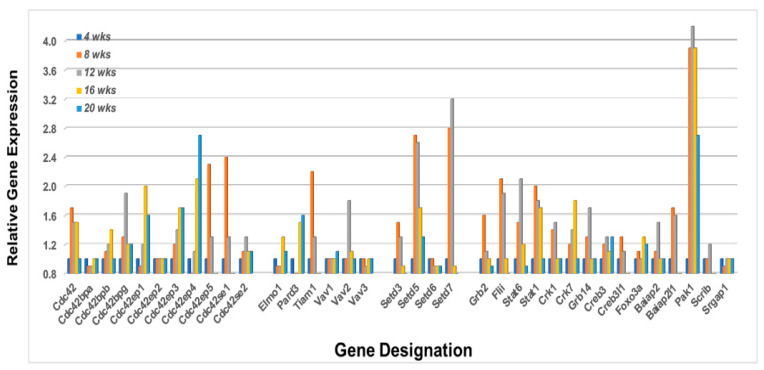
Temporal gene expressions for various proteins important in signal transduction pathways involving the Rho-GTPases. Transcriptome profiles for multiple family genes (e.g., *Cdc42*, *Vav*, *Setd*, *Crkb*, *Stat*, *Crk*, and *Baiap*)), plus individual genes (e.g., *Tiam1*, *Grb2*, *Flii*, *Creb3*, *Foxo3a* and *Pak1*) in the lacrimal glands of C57BL/6.NOD-*Aec1Aec2* mice. Examination of the temporal differential gene expressions, it appears that the heightened transcriptome profile at 8 week is *Cd42 > Cdc42ep5 > Cdc42se1 > Tiam1 > Setd3 > Setd7 > Grb2 > Flii > Stat6 > Stat1 > Crk1 > Greb3> Creb3l1 > Baiap2 > Pak1*, while at 16 weeks it is *Cdc42 > Cdc42bpb > Cdc42ep1 > Cdc42ep4 > Elmo1 > Pard3> Crk7 > Foxo3a > Pak1*.

Lastly, Rho-GTPases are known to interact with numerous proteins that subsequently form active signal transduction pathways capable of regulating multiple activations of bioprocesses critical to cellular functions, especially migrations. These include members of several protein families including Cdc42 interactive proteins, Vav proteins, Bai1-associated proteins, Stat proteins, Forkhead box factors, and various RhoGTPase signaling proteins. A transcriptome profile of these interactive molecules from the lacrimal glands of C57BL/6.NOD-*Aec1Aec2* mice is shown in Figure 9. While most of the genes listed also have roles in multiple signal transduction pathways and cellular functions, an interesting profile is revealed when considering those genes upregulated 8 to 12 weeks of age, in-line with the temporal profiles of upregulated Rho-GTPases in the lacrimal glands presented above. These include *Cdc42*, *Cdc42bpb*, *Cdc42bpg*, *Cdc42ep3*, *Cdc42ep5*, *Cdc42se1*, *Cdc42se2*, *Vav2*, *Tiam1*, *Setd3*, *Setd5*, *Setd7*, *Grb2*, *Flii*, *Stat6*, *Stat1*, *Crk1*, *Crk7*, *Grb14*, *Creb3*, *Creb3l1*, *Foxo3a*, *Baiap2*, *Baiap2l1*, *Pak1*, and *Scrib*. Moreover, the genes *Stat6* and *Stat*1 show temporal upregulated expression profiles consistent with their known functional activities in SS, i.e., Stat6 being critical during early stages of SS and Stat1 being important in later stages of disease, while Pard3 exhibits a later upregulated expression at 20 weeks.

## 3. Discussion

More than two decades ago, studies by Robinson et al. [57] using the NOD-*Igµ*^−/−^ gene knockout (KO) mouse model revealed an absolute requirement for B cells and immunoglobulin in the development and onset of SS-like disease that normally develops spontaneously in parental NOD/ShiLtJ autoimmune mice. Studies in both BAFF [32,33,34] and B6.*Il14α* transgenic (TG) mice [28,29] not only support this earlier finding, but have further shown that elimination of the MZB cell population or blocking the lymphotoxin activity required for MZB cell ontogeny [35] prevents development of SS-like diseases, including lymphomagenesis. MZB cells are an unique subpopulation of bone marrow-derived B lymphocytes characterized by limited expression of immunoglobulin variable region genes that produce predominantly IgM antibodies, many of which are self-reactive [46]. In addition, they are strategically located within mucosal surfaces, function as innate and/or transitional cells capable of rapid responses to both T cell-independent and T-cell dependent antigens, and regulate the activation of subsequent adaptive immune responses by T and B2 lymphocytes in association with monocytic and neutrophilic antigen-presenting cells (APCs) (reviewed in [46]). Functionally, MZB cells differentiate from transitional type-1 (T1) B cells under the influence of low affinity B cell receptor (BCR) signals and transcription factors such as Notch-2, RBP-J and MAML1 [40]. They are enriched within splenic marginal zones (MZs), retained there by interactions between integrin and integrin receptors (e.g., LFA1, ICAM, VLA-4, and SIP1 [48] and respond to Toll-like receptor signaling, especially TLR2, 4 and 9 [15,48,58]. Despite their ability to respond to self-antigens, MZB cells reportedly are more adept at responding to pathogens due to an apparent weak signaling through their BCRs [53,59]. Importantly, MZB cells have been identified within the salivary glands of patients with salivary gland disease, and these infiltrating cells secrete cytokines cytotoxic to salivary gland cells [14,60,61]. Despite these observations and recognized extensive cellular attributes, MZB cells have generally been ignored in studies of SS in both humans and mouse models, in part because MZB cells are a difficult cell population to isolate functionally and then manipulate. However, using temporal global transcriptomic microarrays, we are now able to follow MZB cell markers to identify their temporal presence and potential molecular bioprocesses involved in autoimmune diseases.

Over the past two decades, we have studied multiple murine models of Sjögren’s syndrome to identify the molecular and cellular factors involved in development and onset of those pathophysiological manifestations associated with destruction and loss of lacrimal and salivary gland tissue and subsequent secretory functions. Although it is clear that a T cell-mediated autoimmune process is involved in glandular destruction and onset of clinical disease, actual initiation of SS appears to be fully dependent on B cells [16,57], specifically the MZB cell population per se. This finding raises the question of which molecular bioprocesses activated in MZB cells are relevant to the early underlying stage(s) of SS autoimmunity. The current study addresses two important issues in this regard: (1) is there evidence that MZB cells are involved in the initial immune response(s) that result ultimately in SS disease of the eye, and (2) is the early-stage gene expression profile of lacrimal glands in SS^S^ C57BL/6.NOD-*Aec1Aec2* mice identical to our previously published data for salivary glands [36,37]. Results could clearly impact how future intervention therapies may need to be designed.

MZB cells possess the capability for mounting immune responses to both T-dependent and T-independent antigens, as well as for regulating both innate and adaptive immunity [62]. This capacity results from an inherent interaction between polyreactive BCRs, TLRs, S1P, chemokine receptors, and complement receptors with integrin LFA1 [48,63,64,65]. High LFA1 expression in splenic MZs prolongs retention of MZB cells and reduces their circulation through the blood and lymph systems This retention permits the BCRs of MZB cells direct access to blood-borne antigens presented by dendritic cells (DCs) and granulocytes (GCs). In addition, complement-decorated antigens bind to complement receptors, while an array of antigens can also bind to TLR and S1P receptors. In turn, antigen-activated MZB cells possess the capability to present antigens to T lymphocytes or, with costimulatory signals, differentiate into IgM-secreting plasmablasts that can undergo class switching in the correct environment. Lastly, MZB cells express several chemokine receptors that direct immune cell migrations along chemokine ligand gradients. An important chemokine receptor is Cxcr5 whose ligand is chemokine Cxcl13. At high concentrations, Cxcl13 dislodges MZB cells from MZs, permitting their migration to sites of injury [66]. Transcriptome data presented here indicate a marked upregulation of several chemokine receptor and chemokine ligand, especially *Cxcr5* and *Cxcl13*, proximate with the appearance of infiltrating lymphocytes in the lacrimal glands [38].

MZB cell activation against T cell-independent antigens generally occurs in MZs through presentation of antigens to their TLRs via macrophages, or to their BCRs via reticular cells, neutrophils, and/or dendritic cells. Neutrophils and dendritic cells release both BAFF and APRIL (which in turn activates TACI on MZB cells), while neutrophils also secrete IL-21 which is involved in class-switch recombination and somatic hypermutation that can lead to IgA and IgG synthesis by both MZB cells and T follicular helper cells of germinal centers [67,68,69,70]. Dendritic cells also release chemokine ligands Cxcl9, Cxcl10, and Cxcl11 which bind to chemokine receptor Cxcr3. However, no evidence was seen for upregulated *Il21* or *Cxcr3* gene expressions out to 20 weeks of age in the lacrimal glands of C57BL/6.NOD-*Aec1Aec2* mice, possibly suggesting an absence of B cell class switching per se within the lacrimal gland itself. In contrast, *Cxcr4* and its ligand *Cxcl2*, as well as *Ccr7* and its ligands *Ccl19* and *Ccl21*, are each upregulated, which may point to the initial recruitment, appearance, and activation of the T cell-mediated response.

In contrast to murine MZB cell responses against T cell-independent antigens, murine MZB cells responding towards T cell-dependent antigens can utilize several uniquely different molecular processes. First, Dcir2+ MZ dendritic cells capture and present antigens to both MZB cells and CD4+ T cells. This event promotes the T cell population to differentiate into Th2 cells that secrete Cd40 ligand and IL4, providing an environment for reactive MZB cells to transition to an antigen-presenting cell and differentiate to IgG_1_-producing plasmablasts. However, neither the *Cd40* nor *Cd40* ligand gene exhibited upregulated expression within the lacrimal glands. Second, MZB cells that are lipid-reactive can present various glycolipids via Cd1d molecules to invariant natural killer T (iNKT) cells which, with a concomitant production of IL4-, Cd40 ligand-, and Ifnγ-activated iNKT cells, can induce differentiation of the MZB cells to IgM/IgG plasmablasts. Third, MZB cells that capture complement-opsonized antigens downregulate their S1P receptors, which enhances Ccr5+ MZB cell recruitment by chemokine Cxcl13 to splenic and/or nodal follicles where they provide captured antigen presentation to follicular DCs for activation of follicular T and B cells. While migration of MZB cells is reportedly dependent on LSC (encoded by *Arhgef1*), more recent studies have indicated that Arhgef6 can perform this function as well [71]. Our transcriptomic profiling of lacrimal glands indicates that both *Arhgef1* and *Arhgef6* genes are upregulated, different from salivary glands where only *Arhgef6* is upregulated [37], and this upregulation occurred with highest gene expressions at 12 weeks of age.

Although the microvasculature of mouse and human splenic marginal zones (MZs) are anatomically different, with human spleens apparently lacking a marginal sinus influencing migratory pathways, both species provide environments for MZB cells to interact with APCs in their respective stromal reticular cell networks [72], thereby permitting efficient immune surveillance of blood-borne antigens. The precise cellular functions attributed to MZB cells in autoimmunity and SS clearly require further definition, yet both species separate MZB and follicular B cells via the periarteriolar lymphoid sheaths (PALS). Furthermore, both mouse and human MZB cells tend to utilize similar emigration and activation signaling. For the present study, an overriding question is whether MZB cells function as the inducers of innate autoimmunity [73,74,75]. This requires a correct stromal cell environment within MZ areas and, most likely, the immune-targeted autoantigens to which an extra-nodal lymphogenesis occurs. Ontological development of MZs requires induced expression of ICAM1 (intercellular adhesion molecule-1), VCAM1 (vascular cell adhesion-1), and MADCAM1 (mucosal addressin cell adhesion molecule-1) that, in the presence of IL-7, recruits lymphoid tissue inducer cells (LTIs) that express RXRγt to sites surrounding blood vessels [76]. LTIs secrete lymphotoxin that stimulates secretion of chemokines, especially CCL19 and CCL21, that attract dendritic and lymphoid cells, plus regulate endothelial cells delineating marginal sinuses [77]. Three other stromal cell populations that dictate ontological effects on MZs are the marginal reticular cells (MRCs) which express RANKL (receptor activator of NF-*k*β ligand), fibroblast reticular cells (FRCs) which along with MRCs regulate immune responses via production of reticular fibers and chemokines that guide lymphocyte trafficking, plus follicular dendritic cells (FDCs) which secrete CXCL13 and BAFF to enhance B cell maturation and survival [78]. Importantly, these stromal cell populations secrete type 1 interferon(s) in response to B cell secretion of lymphotoxin, promoting rise of IFN-signatures for autoimmune diseases such as rheumatoid arthritis (RA), systemic lupus erythematosus (SLE), and Sjögren’s syndrome (SS) [6,79]. The current study now provides a possible molecular basis for the prolonged IFN1 production since our study reveals a failure in suppression of *Tbk1* gene expression via the Nlrp4 > Dtx4 pathway thereby prolonging the IFN signal transduction pathway [80].

As stated above, MZB cells are activated through BCR and TLR antigen binding plus TACI binding of BAFF and/or APRIL. While TACI interacts with TLRs to stimulate the MYD88 > TRAF signal transduction pathway that subsequently activates Nf-*k*β, it can also interact with BCRs to activate class switch recombination (CSR) in the presence of CD40 to initiate rapid innate antibody responses [81]. In this regard, murine MZB cells possess strong antigen-presentation capability for CD4+ T cells due in part to elevated expressions on MZB cells of MHC class II, CD80 and CD86, while lipid-activated MZB cells activate invariant natural killer T (iNKT) cells [82,83]. Thus, while MZB cells are strong producers of IgM antibodies, under the correct environment, MZB plasmablasts per se can be highly capable of producing both IgA and IgG antibodies that enhance targeted phagocytosis and killing [84]. Class switch recombination in MZB cells also appears to be regulated by a distinct neutrophil population present in the peri-MZs and characterized by high productions of BAFF, APRIL, CD40L, IL-6, IL21, and chemokines, especially CXCL12 [46]. Although these factors have been reported to be upregulated in various SS^S^ mouse models, the *Il21*, *Cd40*, and *Cd40l* genes were not found to be upregulated in the lacrimal glands of the C57BL/6.NOD-*Aec1Aec2* mice. Overall, these data appear to explain the observations that individuals with neutropenia, defective TACI and/or an impaired STAT3 have reduced MZB cells and exhibit reduced IgA and IgG antibody production toward T-dependent antigens.

Results of the present transcriptomic analysis also reveal distinct temporal gene profiles for Rho-GTPase family and subfamily proteins in the lacrimal glands of our SS^S^-susceptible mice. Rho-GTPases are known to play critical roles in multiple biological processes, especially cell migrations. While our previously published genome analyses [85] identified the *Rac > Ras > Raf > Erk > AP* pathway in development of glandular pathology, the current in-depth analysis of the Rho-GTPases suggests a more complex biological process involving multiple Rho-GTPase and Rho-GTPase-associated gene sets. For example, Tedford et al. [86] reported that Vav1/Vav2-negative B cells are unresponsive to thymus-independent antigens in vivo and indicated a role for Vav-2 in BCR calcium signaling critical to B cell development and function. Similarly, Wang et al. [87] have reported that membrane-proximal BCR signaling molecules (including Vav3, Lyn, Syk, Btk, PLC-γ2, and Blnk) together with adaptor molecules Grb2, Cbl, and Dok3 link BCR micro-clusters and motor proteins. Our transcriptome data indicate that *Tiam1* and *Vav2*, but neither *Vav1* nor *Vav3* are temporally upregulated in the lacrimal glands of C57BL/6.NOD-*Aec1Aec2* mice, while *Pard3*, known for its ability to interact with Tiam to suppress Tiam1-Vav2-Rac1complex function [88], is upregulated concomitantly with the downregulation of Tiam1 and Vav2, thereby establishing a profile suggesting an interesting activation versus regulation of cell differentiation, polarity, division and emigration.

Lastly, a special set of Rho-GEF proteins is the subfamily of DOCK molecules. A direct correlation exists between upregulated DOCK subfamily molecules Dock2, 8, 10, and 11 with cellular homeostasis, development, and migration [89,90,91]. Interactions of DOCK molecules with the Rho-family CDC42 proteins are also known to activate conformational changes in p21-activated PAK family molecules that regulate actin reorganization critical to cell adhesion and invasiveness, as well as upregulation of RAF and RAS pathways leading to activations of ERK, NF-kB, and AP1 (c-Jun, c-Fos and Atf2). As first reported by Namekata et al. [51], expressions of DOCK subgroup D molecules (i.e., Dock9, 10, and 11) are primarily in peripheral blood lymphocytes. However, more recently, evidence suggests that Dock10 expression is uniquely upregulated in B cells by IL-4 and that Dock10.1 isoform regulates T cell activities, while Dock10.2 isoform regulates CD23 expression on B cells, sustains B cell lymphopoiesis in secondary tissues, and up-regulates the IL4 > Stat6 pathway [89]. Our previously published studies have shown that IL4 and Stat6 gene knockout SS^S^ mice prevents SS disease development [24,25]. In addition, *Dock2-Pi3kdelta* activation regulates B cell migration and proliferation, while *Dock2-Pi3kgamma* activation regulates T cell migration and proliferation [90,91]. While the importance of Dock10 cannot be ignored in B cell activation, Dock2 gene knockout mice are deficient in MZB cells [90], as are mice with deficiencies in Rac2, Pyk2 or Rho-GEFs. Nevertheless, both B and T cell emigrations into the exocrine glands of C57BL/6.NOD-*Aec1Aec2* mice occur concomitantly with the upregulation of *Dock2* and *Dock10*.

In summary, data presented from this temporal global transcriptome analysis provide new insights into the role of B lymphocytes, specifically MZB cells, play in development of SS autoimmunity in the lacrimal glands. It identifies probable molecular mechanisms and bioprocesses involving MZB cell emigration to the lacrimal glands during the covert phase of disease development, and the genetic basis for several observed pathophysiologic characteristics of the disease previously identified histologically [38,92,93]. The transcriptome profiles support the presence of a MZB cell population in lacrimal glands of our SS^S^ C57BL/6.NOD-*Aec1Aec2* mouse model beginning as early as 8 weeks of age, a time when autoantibodies, e.g., anti-nuclear autoantibodies (ANA) and anti-muscarinic receptor (M3R) antibodies, are first detected in sera and small peri-ductal lymphocytic aggregates are seen in the lacrimal glands [38]. A Notch2 signal transduction profile in the lacrimal glands suggests a MZB cell population that is undergoing both ontogenesis and activation, with the latter persisting through the early disease stage. Considering the importance of MZB cells in rapid immune responses to disease, it is not surprising to see a transcriptomic profile defining MZB cells and one that changes temporally. In conjunction with our previously published studies indicating absolute requirements for Ig BCRs [57], C’3 [26], IL4 [24], STAT6 [25], T_H_17 cells [27], and IFN [23] in the development of an overt clinical disease, we propose that the transition to a T cell-mediated cytotoxic response most likely occurs in the follicular areas of the spleen and lymph nodes induced by MZB cells emigrating from the exocrine glands back to secondary lymphoid tissues. These 8- and 16-week timepoints, therefore, represent potential focal points for intervention therapy if diagnosed in time. Nevertheless, it is important to note that our transcriptome analyses strictly reflect the ongoing bioprocesses in the lacrimal glands and fails to address any active bioprocesses occurring in the follicles of the draining lymph nodes or spleen [94]. Nevertheless, the current study shows how the gene profile described here for lacrimal glands mimics closely our published gene profile for salivary glands, at the same time reveals important differences. Furthermore, the study also demonstrates the power of temporal transcriptomic analyses to establish a foundation for further in-depth investigations that are expected to identify uniquely novel targets for possible testing of intervention therapies.

## 4. Materials and Methods

### 4.1. Animal Model

C57BL/6.NOD-*Aec1Aec2* mice, a diabetes-free model of primary SS-like disease, were bred and maintained under specific pathogen free (SPF) conditions within the College of Medicine’s Department of Pathology’s Mouse Facility with oversight by Animal Care Services at the University of Florida, Gainesville. The C57BL/6.NOD-*Aec1Aec2* mouse is a well-studied and well-characterized model of SS that spontaneously develops features of SS observed in patients. Mice were maintained on a 12-hr light–dark schedule and provided food and acidified water ad libitum. Any mice showing signs of eye dryness were treated daily with salve ointment. For the present study, breeding pairs (one male and one female) were paired per cage and offspring were weaned at 3 and 5 weeks of age. Weaned mice were caged maximum of *n* = 5 per cage per sex. The experimental mice were raised to a maximum of 20 weeks of age, a time prior to signs of any overt or covert disease. Euthanization was carried out at the appropriate timepoints by cervical dislocation after deep anesthetization as stipulated by the Panel on Euthanasia of the American Veterinary Association. Lacrimal and salivary glands were explanted simultaneously from the same animals. There were no indications that this procedure affected subsequent preparation of RNA specimens. Both the breeding and use of these animals for the present studies were approved by the University of Florida’s Institutional Animal Care and Use Committee (IACUC) under protocols 2008011756 and 201004828.

### 4.2. RNA Preparations

Procedures for the isolation, preparation, and quality testing of RNA samples are described in detail elsewhere [92,93]. In brief, extra-orbital lacrimal glands, free of lymph nodes, were excised in parallel from male C57BL/6.NOD-*Aec1Aec2* mice euthanized at 4-, 8-, 12-, 16-, or 20 weeks of age (*n* = 4–6 per age group), snap-frozen in liquid nitrogen and stored at −80 ˚C until all samples were obtained. Using one lacrimal gland from each mouse, total RNA from each specimen was isolated concurrently using the RNeasy Mini-Kit (Qiagen, Valencia, CA, USA), as per the manufacturer’s recommended protocol. Each RNA sample was hybridized on an Affymetrix 3′ Expression Array GeneChip Mouse Genome 430 2.0 array and annotated (build 32; 06.09.2011). Each GeneChip contained 45,102 DNA sequences, thus the resulting data set consisted of 1,127,550 data points. A heatmap of differentially expressed genes is published elsewhere [92]. To verify relative microarray gene expressions, numerous genes were randomly selected for relative comparative expression by real-time polymerase chain reaction (rt-PCR) analyses (see [92]. The rt-PCR data confirmed both differential and temporal gene expressions. Full microarray data libraries are deposited with Gene Expression Omnibus, Accession number GSE15640.

### 4.3. Microarray Data Analyses

Gene expression analyses have been detailed elsewhere [92,93]. In brief, microarray data were normalized using the robust multiarray average (GCRMA) algorithm, followed by Linear Models for Microarray Analysis (LIMMA) (http://www.r-project.org) (accseesed on 6 April 2022) for differential expression determinations. The fdr (false discovery rate) method of Benjamini and Hochberg [95] was used to adjust the *p*-values for multiple testing. The original data represent 5 equally spaced timepoints; thus, multiple models were used to identify temporal patterns of gene expression, i.e., the linear fit (degree = 1), quadratic fit (degree = 2), cubic fit (degree = 3), and quartic fit (degree = 4) regression models. At this point, each gene has a single statistical value based on *n* = 5 data points at each of the 5 timepoints. B-statistics were calculated for each gene providing odds that a gene shows either positive or negative trends over time. In addition, these temporal changes in an individual gene’s expression relative to its value at the 4 weeks timepoint were considered; thus, data presented show the differential expression of an individual gene’s value at the 8, 12, 16 or 20 weeks timepoints relative to that gene’s value at the 4 weeks timepoint set as 1.0. The 4 weeks’ time point is a time when the exocrine glands have reached functional maturity and considered in a pre-diseased state. In the current study, the gene selection is not based on a SS-non-susceptible (SS^NS^) mouse strain, but on data generated in similar studies carried out using the salivary glands from the C57BL/6.NOD-Aec1Aec2 SS-susceptible (SS^S^) mouse strain; thus, our previously published gene expression data from the salivary glands represent the comparative data [69].

## 5. Conclusions: Summary and Contribution to the Field

Sjögren’s syndrome (SS), a human systemic rheumatoid autoimmune disease affecting lacrimal and salivary gland functions that lead respectively to dry eye and dry mouth pathologies, is considered an under-studied disease appearing most frequently in middle-aged women. Patients generally present in clinic only after the adaptive autoimmune phase is active and irreversible pathology has occurred. Furthermore, the time between disease onset and a correct diagnosis can range from 4 to 10 or more years, permitting the pathology to progress unchecked without intervention therapy, plus seriously slowing generation of effective therapy development. To address these issues, numerous mouse models exhibiting SS-like disease have been developed that are useful in identification of pre-disease phases of the autoimmune processes. While studies in these rodent models have addressed multiple cellular and pathological events, many details of the underlying cellular and molecular events remain unidentified. One observation that has recently come to light is an early-stage appearance of MZB cells within the salivary glands occurring concomitantly with the onset of SS pathology. Here, we provide evidence that indicates MZB cells are also infiltrating the lacrimal glands of the C57BL/6.NON-Aec1Aec2 mouse model early in the development of its SS-like disease. Furthermore, we have begun to define molecular processes involved with the cellular emigration to the lacrimal gland, specifically temporal activations of genes representing chemokines, focal adhesions, and Rho-GTPases. Comparisons of the present lacrimal gland profiles with those previously published for the salivary gland [36,37] indicate both similarities and differences in molecular processes occurring in the two glands. In summary, using temporal global transcription analyses, we have begun identifying molecular profiles for those critical bioprocesses involved in SS development and onset; however, the different profiles emerging for salivary and lacrimal glands are suggesting potential autoimmune processes that could seriously impact how one approaches design and development of potential therapies.

## Data Availability

Gene Expression Omnibus, Accession number GSE15640.

## Abbreviations

APC	Antigen-presenting Cell
APRIL	Tnfsf13 (Tumor necrosis factor superfamily, member 13
ARHGAP	Rho GTPase activating protein
ARHGEF	Rho GTPase exchange factor
BAFF	Tnfsf13b
BCR	B cell receptor
B220	CD45-receptor
CCR	Chemokine (C-C motif) receptor
CCL	Chemokine (C-C motif) ligand
CD	Cell differentiation antigen
CDC42	Cell division cycle 42
CXCL	Chemokine (C-X-C motif) ligand
CXCR	Chemokine (C-X-C motif) receptor
DC	Dendritic cell
DOCK	Dedicator of cytokinesis
FACS	Fluoresec-activated cell sorting
GC	Granulocyte
GCRMA	Guanine-cytosine robust multi-array average
GTP-GAP	G protein bound-guanine nucleotide activating protein
GTP-GEF	G protein bound-guanine nucleotide exchange factor
HDAC	Histone deacetylase complex
ICAM1	Intercellular adhesion molecule 1
IFN	Interferon
IL	Interleukin
KO	Knock-out (gene)
LIMMA	Linear models for microarray analysis
LF	Lymphocytic foci
LFA1	Lymphocyte function-association antigen 1
LTI	Lymphoid tissue inducerr cell
MADCAM1	Mucosal cell adhesion molecule-1
MZ	Marginal zone
Mapk	Mitogen-activated protein kinase
MRC	Marginal reticular cell
MZB	Marginal zone B cell
NF-kb	Nuclear factor-kb
NK	Natural killer (cell)
NOTCH	Notch gene homolog
PALS	Periarteriolar lymphoid sheaths
PTK2	Protein tyrosine kinase
RAC	Ras-related C3 botulinum toxin substrate
RANKL	Receptor activator of NF-kb
rt-PCR	Real-time polymerase chain reaction
RA	Rheumatoid arthritis
RHO-GTP	Ras homolog member
RNA	Ribonucleic acid
RND	Rho-family GTPase
RSF	Remodeling and spacing factor
SLE	Systemic lupus erythematosus
SS	Sjögren’s syndrome
S1P	Sphingosin-1-phosphate
S1PR	Sphingosine-1-phosphate receptor
TG	Transgenic
TLR	Toll-like receptor
VCAM1	Vascular cell adhesion molecule 1
VLA4	Very late antigen 4
